# Anti-Tumor Immunity in Head and Neck Cancer: Understanding the Evidence, How Tumors Escape and Immunotherapeutic Approaches

**DOI:** 10.3390/cancers7040900

**Published:** 2015-12-09

**Authors:** Clint T. Allen, Paul E. Clavijo, Carter Van Waes, Zhong Chen

**Affiliations:** 1 Head and Neck Surgery Branch, National Institute on Deafness and Other Communication Disorders, National Institutes of Health, Bethesda, MD 20892, USA; clint.allen@nih.gov (C.T.A.); paul.clavijo@nih.gov (P.E.C.); vanwaesc@nidcd.nih.gov (C.V.W.); 2 Department of Otolaryngology-Head and Neck Surgery, Johns Hopkins School of Medicine, Baltimore, MD 21287, USA

**Keywords:** syngeneic mouse models, tumor antigen, innate immunity, adaptive immunity, immunosuppression, immunogenicity, antigenicity, checkpoint inhibitors, vaccines, immunotherapy

## Abstract

Many carcinogen- and human papilloma virus (HPV)-associated head and neck cancers (HNSCC) display a hematopoietic cell infiltrate indicative of a T-cell inflamed phenotype and an underlying anti-tumor immune response. However, by definition, these tumors have escaped immune elimination and formed a clinically significant malignancy. A number of both genetic and environmental mechanisms may allow such immune escape, including selection of poorly antigenic cancer cell subsets, tumor produced proinflammatory and immunosuppressive cytokines, recruitment of immunosuppressive immune cell subsets into the tumor and expression of checkpoint pathway components that limit T-cell responses. Here, we explore concepts of antigenicity and immunogenicity in solid tumors, summarize the scientific and clinical data that supports the use of immunotherapeutic approaches in patients with head and neck cancer, and discuss immune-based treatment approaches currently in clinical trials.

## 1. Introduction

Enhanced understanding of the underlying mechanisms behind control of the development and progression of malignancies by the immune system has led to the general acceptance of immune-based treatments as being a viable approach to treat cancer and the development of new immunotherapeutic approaches. While murine models provided much of the preclinical hypothesis generating data, many of these concepts are being validated in retrospective studies of human tissues following treatment with immune-targeting agents and in prospective clinical trials. Head and neck squamous cell carcinoma (HNSCC) has been intensely studied, both because of its poor prognosis, need for enhanced treatment options and relative ease of tissue acquisition compared to other solid tumor types. One decade ago, the overwhelming majority of HNSCC clinical trials were designed to investigate targeted therapies with the goal of blocking an oncogenic “driver” signaling pathway within the cancer cell itself. While this was a valid approach and remains so today, issues such as tumor heterogeneity and multiple resistance mechanisms following single pathway inhibition have limited the durable responses observed. While oncogenic signaling within a cancer cell can contribute to a poorly immunogenic tumor microenvironment, immune recognition and subsequent elimination of a cancer cell fundamentally is independent of underlying driver mutations. We are only now beginning to understand the importance of factors such as mutational load, genomic instability and intracellular oncogenic signaling. Today, the majority of clinical trials being performed across the country are immunotherapy based. In this review, we summarize early preclinical work that initially led to the recognition that deregulated immune responses were important factors in the tumorigenesis of HNSCC and how knowledge generated using other solid tumor models has led to a firm understanding of why some HNSCCs are able to escape anti-tumor immunity. We also systematically review many of the immunotherapy approaches currently being investigated.

## 2. Early Evidence that Deregulated Immunity Plays a Role in HNSCC Progression in Preclinical Models

To establish a preclinical model to study immunologic events associated with squamous carcinoma progression, the Pam 212 model was established by subcutaneously transplanting cells that spontaneously transformed following long term culture of neonatal keratinocytes [1]. These parental tumors were not highly-immunogenic as they did not regress when transplanted into syngeneic BALB/c mice [2]. Rare metastatic Pam 212 variants following serial subcutaneous transplantation into BALB/c and nude mice were isolated and cultured *in vitro* [3]. When transplanted back into BALB/c mice, these metastatic Pam-LY (from lymph node metastasis) and Pam-LU (from lung metastasis) variants demonstrated aggressive primary tumor growth and frequent spontaneous metastasis. No difference in *in vitro* growth rates between the parental Pam 212 and metastatic variant lines suggest a host-dependent mechanism that was independent of adaptive immunity, as similar findings were observed in BALB/c SCID mice. Characterization of oncogenic signaling within the parental and metastatic variants revealed increased NF-κB activity and expression of downstream proinflammatory cytokines interleukin (IL)-1, IL-6, granulocyte/monocyte-colony stimulating factor (GM-CSF) and CXCL1 [4,5,6]. Stable transfection of a CXCL1 expressing vector into parental Pam 212 lines recapitulated the aggressive primary tumor growth and metastatic phenotype of the metastatic variant lines, which demonstrated enhanced myeloid and monocyte leukocyte infiltration into the tumor microenvironment. This aggressive phenotype was attenuated in CXCR2 knockout mice, mechanistically linking enhanced NF-κB activity, CXCL1 expression, CXCR2-dependent leukocyte recruitment into the tumor microenvironment and aggressive *in vivo* phenotype [7,8,9,10].

To further characterize the link between NF-κB driven expression of proinflammatory cytokines and deregulated systemic immunity, parental Pam 212 or metastatic variant cells were transplanted into syngeneic mice and Th1 cytokine mediated delayed-type hypersensitivity (DTH) was measured [11]. Mice bearing metastatic variant tumors demonstrated significantly decreased DTH reactions compared to mice bearing parental Pam 212 tumors. Further, significant megalosplenia, which developed in mice bearing metastatic variant tumors, was found to be due to increased accumulation of Gr1^+^CD11b^+^ immature myeloid cells. Characterization of cytokine expression patterns in these accumulated myeloid splenocytes in tumor bearing mice revealed a Th2 dominant pattern with decreased IL-2, IL-12, interferon (IFN)-γ and tumor necrosis factor (TNF)-α and elevated IL-4 and transforming growth factor (TGF)-β. When transplanted into IL-4 deficient mice, both parental Pam 212 and metastatic variant tumors demonstrated suppressed tumor growth [11]. These studies were among the first to firmly establish a link between oncogenic cytokine signaling, the development of deregulated host immunity, and malignant progression in SCC.

To explore whether similar links between oncogenic signaling and the development of dysfunctional anti-tumor immunity could be established in a carcinogen-induced SCC cells transformed *ex vivo*, lingual keratinocytes were transformed *in vitro* using 4-nitroquinolone-1-oxide. Following tumor development in immune-deficient mice, multiple cells lines that either rejected (regressors) or grew progressively (progressors) when transplanted into immune competent mice were established [12]. Regressors were found to express the B7 family co-stimulatory protein CD80, whereas progressors lacked CD80 expression. This dichotomy of CD80 expression was found to be critical in the anti-tumor response to systemic IL-12 and peritumoral IL-2 immunotherapy, as tumor generated from cell lines lacking CD80 expression failed to respond [13]. Regression of CD80^+^ tumors following this immunotherapy regimen was abrogated in IFNγ deficient mice, and 50% of mice who had complete regression of CD80^+^ tumors rejected tumor transplantation upon re-challenge, firmly establishing an immune mechanism. While CD80 expression could be restored by IFNγ treatment, NF-κB dependent cytokines IL-1, IL-6 and GM-CSF suppressed CD80 expression in progressor cell lines [14], once again linking oncogenic signaling with the development of local immune dysregulation.

More recent work has linked not only aberrant NF-κB signaling with chemotactic cytokine expression from SCCs, but has also highlighted the role of the TP63 family member ∆Np63. Originally hypothesized to be playing a role in SCC pathogenesis due to its location within a commonly amplified locus in patients with HNSCC (3q28) [15], ∆Np63 physically interacts with the NF-κB family member c-Rel to form a transcriptional complex that drives expression of IL-8, in human HNSCC cells [16,17,18]. Using a transgenic mouse model that allows inducible over-expression of ∆Np63, tissues overexpressing this transcription factor expressed CXCL1, the murine homolog of IL-8, demonstrated robust myeloid cell (CD11b^+^) and T-regulatory cell (T_reg_; CD4^+^CD25^+^FoxP3^+^) infiltrates, similar to Pam-LY cells [19]. Clearly, preclinical evidence supports that the concept oncogenic and proinflammatory signaling within HNSCC cells contributes to the recruitment of suppressive immune cells within the tumor microenvironment.

Over the last decade, pioneering work by many other labs using various solid tumor models has firmly established the role of dendritic cells, type I (IFNα and β) and II (IFNγ) interferon and T-lymphocytes in the cross presentation of tumor antigens and development of antigen-specific adaptive immune responses against malignant cells [20,21,22]. This has led to a general acceptance of the critical role that the natural immune response plays in controlling both the development and progression of malignancies. Indeed, evasion of host immunity has been added as a critical feature of malignant development and progression in Hannahan and Weinberg’s “Hallmarks of Cancer [23]”.

## 3. Evidence that the Immune System Limits Formation and Progression of Human HNSCC

Many patients with cancer, including HNSCC, demonstrate measureable tumor antigen specific T-cells both peripherally in circulation and within the tumor microenvironment [24,25]. These antigen-specific T-cells can be specific for tumor-associated antigens (TAAs) such as viral epitopes (HPV-derived peptides, for example) or proteins overexpressed in malignant compared to normal cells such as wild-type p53 or mucin 1 [25]. Normally expressed in tissues during fetal development, germline cell products such as carcinoembryonic antigen and MAGE family proteins can be significantly expressed by malignant cells and represent a higher degree of tumor cell specificity when targeted by the adaptive immune system [25]. For many years, the lack of identifiable antigens that were truly specific for an individual cancer cell (TSA, tumor specific antigens) provided for some a rational explanation for why clinically significant tumors were able to evade immune elimination. However, recent evidence has definitively demonstrated that neoantigens, or peptide products from tumor-specific mutated genes, can serve as truly cancer-specific antigens [26,27,28]. In general, the more somatic mutations a cancer cell carries, the more neoantigens it may express [29]. These neoantigens are nearly universally derived from passenger mutations (as opposed to driver mutations that the cancer cell relies upon for growth and survival), opening up the possibility that these neoantigens could be “lost” in the process of the immune system selectively eliminating tumor cells that display strong antigens and leaving behind tumor cells that do not—a process termed immunoediting [30,31]. Of interest, the character of the antigenic peptides also appears to impact the development of meaningful anti-tumor T-cell responses. Peptide antigens that mimic viral and bacterial antigens in amino acid sequence similarity induce more robust anti-tumor immunity [32]. This, along with the deciphered role of type I interferon in tumor antigen presentation, likely explains why Professor Coley was able to induce durable tumor control in a subset of patients following intra-tumor bacterial exposure so many years ago [33].

The concepts of antigenicity and immunogenicity are important to clarify. Antigenicity refers to the ability of a given peptide inside a cell to be bound and presented via MHC molecules on the surface of the cell and binds a T- or B-cell receptor. Immunogenicity then refers to the ability of that peptide:MHC and T- or B-cell interaction to activate an adaptive immune response. Antigenicity is required but not sufficient for immunogenicity as the later requires a complex system of cell types and cytokines and functionally is determined by the summation of many activating and inhibitory signals. Antigenicity is difficult to measure in a human tumor without some *a priori* knowledge of what the potential tumor antigens could be. Surrogate measures of immunogenicity are easier to quantify and include, in its most basic form, the presence of tumor infiltrating lymphocytes (TILs) within a tumor [24]. Using the presence of TILs as a measure of immunogenicity, many HNSCCs are immunogenic. With some variation depending on whether genomic or proteomic approaches were used, about 50% of carcinogen-associated HNSCCs, and a higher proportion of HPV-associated HNSCCs, demonstrate CD8^+^ TIL infiltration [25,34,35]. Analysis of TCGA data by Keck *et al.*, supports that a significant subset of HNSCCs demonstrate a gene expression profile consistent with elevated CD8^+^ TIL presence and activation [35]. HNSCCs demonstrating a higher number of TILs respond better to definitive chemoradiotherapy [36,37] and have better outcomes following surgery with adjuvant therapy [38]. That HPV-associated HNSCC demonstrates in general a higher degree of immunogenicity is not surprising; the observation that virally-induced malignancies are more likely to induce deregulated immune responses than sporadic or carcinogen-induced cancer was made decades ago [39].

## 4. Immune Escape in Solids Tumors and HNSCC

**Figure 1:**
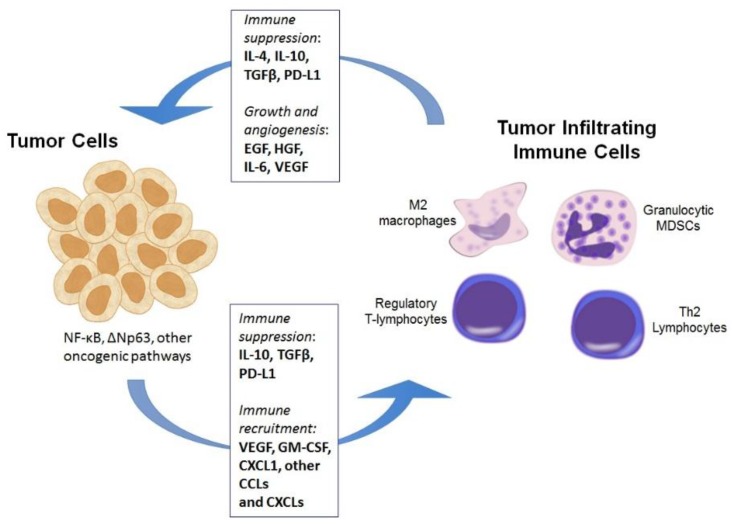
Illustration of many of the mechanisms by which tumor cells, through deregulated oncogenic signaling pathways, induce the infiltration of different suppressive immune cells subsets into the tumor microenvironment. These include M2 (pro-tumor) macrophages, myeloid derived suppressor cells (MDSCs), regulatory T-lymphocytes and Th2 polarized CD4 T-lymphocytes. Many of these immune cells, in turn, directly suppress immune responses via cytokine production and release of immune-modulating enzymes. MDSCs within the tumor microenvironment can also contribute directly to tumor cell growth and survival via the secretion of cytokines and growth factors. While both tumor cells and immune cells can autonomously express checkpoint ligands such as PD-L1 downstream of oncogenic signaling pathways, this appears to be largely interferon responsive in HNSCC and serves to induce “adaptive resistance” in immunogenic tumors with high baseline interferon levels.

If significant portions of patients with HNSCC have tumors that are immunogenic (at least by TIL analysis), why do they have cancer? Why did the visualized immune response present within their tumor not prevent the development of their disease, and why does it not limit its progression? Indeed, all clinically significant tumors have, by definition, escaped immunity [23]. Cancer cells escape immune elimination by a number of mechanisms. These can broadly be characterized as problems with inducing the development of an anti-tumor immune response *vs.* suppression of an activated anti-tumor immune response. Several of these mechanisms are summarized in Figure 1.

### 4.1. Lack of Development of an Anti-Tumor Immune Response

Some tumors may be poorly antigenic from their development. In other words, the cancer cells within these tumors may express insufficient or no TSA or TAA, possibly secondary to a very low mutational rate or sheer chance that the mutated genes they carry produce proteins that are not efficiently processed and/or loaded onto MHC molecules for immune presentation [24]. Alternatively, the theory of immunoediting, as described above, suggests that cancer cells presenting TAAs or TSAs that strongly activate immune responses will be eliminated early in tumor development [31]. Given the high degree of heterogeneity in a complex tumor [40], not all cancer cells within a tumor express the same antigens. Those cancer cells that display weak antigens or no antigen at all will be selected for [30], and unless genomic instability or other alterations lead to the expression of new neoantigens, that tumor will likely have suppressed immunogenicity and escape immune elimination. A separate issue is that of the development of T-cell tolerance to specific TAA or TSA. Experimental evidence supports that chronic exposure to antigen can, in certain conditions, lead to unresponsiveness of T-cells specific for that antigen [41,42,43]. Even in tumors that contain cancer cells that express strong antigens, T-cell exposure in the pre-malignant phase, without the required positive co-stimulatory signals, may lead to T-cell tolerance and immune escape [41].

A related issue is activation (or lack thereof) of innate immunity in the setting of a developing malignancy. Type I interferon signals are specifically required for the cross presentation of tumor antigen by dendritic cells to both CD4 and CD8 T-cells to allow development of an adaptive immune response [21,22]. The expression of type I interferon in the local microenvironment is the end result of signaling downstream of pattern recognition receptors (PRRs; toll-like receptors and STING, for example) and is abundant in the setting of a foreign pathogen, yet can be absent in a developing malignancy [44,45]. Ligands for these innate PRRs include many bacterial and viral products, but also damage associated molecular patterns (DAMPs) that can be released from damaged and/or dying tumor cells [46]. The absence of such signals required to activate innate immunity likely represents a significant barrier in the development of tumor antigen-specific adaptive immune responses, and approaches to enhance so called “sterile inflammation” are a major research focus. The interplay of innate immune signaling in the HNSCC tumor microenvironment is complex, as PRR signaling is required to initiate innate immunity but can also drive tumorigenesis when PRRs are expressed on HNSCC tumor cells [47].

A separate but functionally similar problem is the tendency of cancer cells to down-regulate the expression of proteins required to allow immune cells to detect cancer cell surface antigen. This has been elegantly reviewed by Ferris *et al.* [48]. Briefly, two positive signals are required to activate T-cells to expand and develop effector functions. “Signal one” comes from the interaction between the T-cell receptor on the surface of a T-cell and an MHC molecule presenting an antigenic peptide on the surface of a cancer cell. Specific cytoplasmic proteins (cumulatively called the antigen processing machinery) break down parent proteins into peptides of specific lengths, process these peptides, and load them onto MHC molecules for presentation on the cell surface. Cancer cells within HNSCCs often down-regulate the expression of these components, including TAP-1/2 and MHC class I, compared to normal tissues, effectively blocking MHC:peptide cell surface translocation and subsequent TCR interaction. In addition, positive costimulatory molecules present on the surface of cancer cells required for a positive “signal 2” during cancer cell:immune cell interactions are often downregulated [12]. Evidence indicates that decreased expression of these critical immune processing and signaling components is not due to genetic alterations, but rather due to proinflammatory signaling within the tumor microenvironment [14,48]. Of note, many of these deficits can be recovered or restored upon cancer cell exposure to interferon, suggesting they functionally may not impede the ability of cancer cells expressing TAAs or TSAs to be eliminated by the immune system within a therapeutically optimized tumor microenvironment.

### 4.2. Suppression of Activated Immune Responses

Given the evidence that most if not all tumors express neoantigens capable of inducing a tumor-specific immune response [24] and that most patients with HNSCC demonstrate evidence of measurable immunogenicity, most patients with HNSCC likely do develop an anti-tumor immune response that is subsequently altered or blocked to become ineffective. This has been termed *immunosubversion* [49]. There are physical and microenvironmental barriers that immune cells must overcome to infiltrate into the tumor. First, interstitial pressure within a solid tumor builds from the net effects of cancer cell proliferation, permeable vasculature and a lack of patent lymphatic vessels [50]. This pressure can physically prevent immune cell infiltration and contact with their targets within a solid tumor [51]. The highly abnormal nature of solid tumor vasculature that contributes to high interstitial pressures can itself lead to the inability of immune cells in circulation to reach different geographic regions of the tumor [52]. Additionally, the hypoxic tumor microenvironment potently suppresses immune cell function, and many solid tumors display significant regions of hypoxia depending upon tumor vascularization [53]. Interestingly, therapies that *normalize* tumor vasculature actually make the tumors more susceptible to immune activating treatments [54].

If immune cells are able to overcome the physical barriers above and reach the tumor interstitium, a number of immunosuppressive factors within the tumor microenvironment may limit their function. First, cancer cells may directly express cytokines, such as TGF-β and IL-10, which are directly immunosuppressive [25,55]. These cytokines potently suppress T-cell proliferation and cytotoxic function and are part of the normal checks-and-balances program present within the immune system to prevent uncontrolled cytolytic immune cell function. HNSCC also secrete many chemokines, such as CCLs, CXCLs, and VEGF, that drive the recruitment of many immunosuppressive hematopoietic cells into the tumor microenvironment [56]. The most extensively studied of these immunosuppressive hematopoietic cells includes immature myeloid cells, also known as myeloid derived suppressor cells (MDSCs) [57]. CXCR2^+^ and/or CCR2^+^ MDSCs are recruited to the tumor microenvironment by at least GM-CSF, CXCL1 and IL-8 chemotaxis [58,59,60]. Increased nuclear localization of ∆Np63 and associated NK-κB family member c-Rel correlated with enhanced immune infiltrates in HNSCC specimens [18], linking tumor-derived factors to the recruitment of these immunosuppressive cells as described previously. MDSCs are characterized by the expression of CD11b^+^ and GR-1^+^ in mice and CD11b^+^CD14^+^CD33^+^HLA-DR^−^ in humans [57,61]. Once in the tumor microenvironment, MDSCs are functionally programmed to be immunosuppressive by the local cytokine profile, with Th2-type cytokines IL-4 and IL-10 driving MDSCs to suppress effector T-cells [62]. Elimination of MDSCs from the tumor microenvironment using genetically altered mice, therapeutic antibody depletion, or chemokine blocking antibodies promotes accumulation of effector immune cells within the primary tumor, reduces primary tumor growth and sensitizes tumors to immune-activating therapies in multiple solid tumor types [58,63,64,65,66]. Human HNSCCs are massively infiltrated with MDSCs [61,67,68] that potently suppress local T-cell function via production of arginase (Arg), nitric oxide synthase (NOS), and potentially idoleamine 2,3-dioxygenase (IDO1) [61,67,69]. Functional inhibition of Arg and NOS in MDSCs with phosphodiesterase 5 inhibitors augments tumor specific immunity in patients with HNSCC [67]. Similar to MDSCs, mature macrophages demonstrate functional plasticity and can be polarized into either anti-tumor M1 or pro-tumor M2 phenotypes, again dependent upon the tumor microenvironment cytokine profile [70]. M1 macrophages, when activated by IFN from natural killer cells, possess the ability to limit tumor growth *in vivo* in the absence of adaptive immunity [71]. A majority of macrophages in HNSCC tumors are M2 phenotype and express immunosuppressive cytokines TGFβ and IL-10 [72]. CD4^+^ T-cells can be functionally polarized by the cytokine milieu present in the tumor microenvironment as well. Whereas anti-tumor Th1 cells limit tumor progression by enhancing cytotoxic T-cell responses, pro-tumor Th2 cells skew adaptive responses toward humoral immunity via production of cytokines such as IL-4 and IL-10 [25,73]. FoxP3^+^ regulatory CD4^+^ T-cells (T_reg_s) are another subset of functionally immunosuppressive hematopoietic cells that infiltrate the HNSCC tumor microenvironment and suppress effector T-cells via a number of mechanisms [74,75]. Peripheral and tumor infiltrating T_reg_s are increased in HNSCC tumors compared to normal tissues, T_reg_s accumulate early in malignant progression, and tumor infiltrating T_reg_s are significantly more immunosuppressive compared to their peripheral counterparts suggesting functional reprogramming in the tumor microenvironment [74,76,77,78]. Whether functionally mature Tregs are recruited into the tumor microenvironment via cancer cell secreted chemokines or whether FoxP3 negative CD4^+^ T-cells are functionally converted into FoxP3^+^ T_reg_s by the cytokine milieu present within the tumor microenvironment is still controversial [74].

Though it is clear that HNSCCs recruit immunosuppressive hematopoietic cells into the tumor microenvironment, some debate exists about whether these cells are directly responsible for the lack of T-cell mediated tumor control in otherwise immunogenic tumors, or whether cancer cell immune escape is due to T-cell tolerance of TAA or TSA that develops early in malignant progression [41,79]. One potential mechanism for the induction of tolerance to antigen on the surface of cancer cells is the activation of negative co-stimulatory receptors expressed on tumor infiltrating T-cells. In the “signal 1/2” paradigm to conceptually understand T-cell activation, different receptors on the surface of T-cells can confer a positive or negative “signal 2” [80]. So-called checkpoints are negative co-stimulatory receptors that, when activated by their cognate ligands, functionally suppress T-cell function and can even induce T-cell apoptosis [81]. Normally present to prevent dysregulated immune activation and autoimmunity, tumors have usurped this mechanism of T-cell evasion and express checkpoint ligands, such as programmed death ligand 1 (PD-L1), to suppress T-cells in the tumor microenvironment [82]. While tumor cells themselves can express checkpoint ligands such as PD-L1 downstream of oncogenic signaling, PD-L1 expression appears to be mainly IFN-responsive within the tumor microenvironment [83,84]. Functionally, this means that an immunogenic tumor, which has abundant type I and II IFN present within the microenvironment, will express PD-L1 and evade T-lymphocyte mediated killing of tumor cells (so called “adaptive resistance”). The most heavily studied checkpoints include cytotoxic T-lymphocyte protein 4 (CTLA4) and programmed cell death 1 (PD1), though many others exist and are the study of intense research [85]. Antibodies that bind and block these checkpoints or their ligands induce anti-tumor T-cell activity and durable anti-tumor immune responses in subsets of patients with advanced cancer [86,87]. Promising studies over the last five years have led to the FDA-approval of anti-CTLA4 and anti-PD1 checkpoint inhibitors in multiple solid tumor types, including melanoma and lung cancer [88,89]. Compared to other forms of immunotherapy such as adoptive immune cell transfer of *ex vivo* expanded immune cells, which are much more cumbersome and limited to only a few institutions, the use of checkpoint inhibitors has made the widespread use of anti-tumor immunotherapy practical and broadly applicable. These approaches are discussed below.

## 5. Approaches Utilized to Activate Anti-Tumor Immunity in Patients with HNSCC

Backed by extensive preclinical data mainly in syngeneic murine models of carcinoma, many approaches to enhance anti-tumor immunity in HNSCC are currently being investigated. These approaches, along with trials currently enrolling patients with HNSCC, are summarized in Table 1.

### 5.1. Targeting Immunosuppressive Cells—Myeloid Cells

Approaches designed to functionally inhibit or deplete MDSC from the HNSCC tumor microenvironment are attractive since MDSC are likely to both directly induce HNSCC cancer cell growth and survival through secreted growth factors and immunosuppression as described above. Recent reports by Weed and Califano *et al.* have established that the phosphodiesterase 5 (PDE5) inhibitor tadalafil treatment reduces the number of peripheral MDSCs and enhances antigen-specific T-lymphocyte reactivity in patients with HNSCC [67,90]. Given these promising results, a phase II combining tadalafil with standard of care treatment in HNSCC is currently underway. Several CXCR2 blocking antibodies and agonists are currently being evaluated for safety and efficacy in patents with advanced cancer and pulmonary inflammatory disorders [91]. One early phase trial investigating the possibility that CXCR2 mAbs, designed to block infiltration of MDSCs, can enhance responses to single checkpoint inhibitor in patients with metastatic HNSCC is underway.

**Table 1:** Immunotherapy clinical trials in the United States currently open and enrolling patients.

Drug Category and Name	Mechanism	Combination	Status	Clinical Trial ID	Target Population
Checkpoint/ co-stimulatory studies					
Nivolumab	PD1 blocking mAb	single agent	Phase III	NCT02105636	Recurrent or metastatic HNSCC
Nivolumab	PD1 blocking mAb with CD27 agonist mAb	with Varlilumab	Phase I/II	NCT02335918	Advanced solid tumors
Nivolumab	PD1 blocking mAb with IDO1 inhibitor	with INCB24360	Phase I/II	NCT02327078	Advanced solid tumors
Nivolumab	PD1 blocking mAb with CSF1R blocking mAb	with PLX3397	Phase I	NCT02526017	Advanced solid tumors
Nivolumab	PD1 blocking mAb	single agent	Phase I/II	NCT02488759	Advanced and metastatic HPC-associated HNSCC
Pembrolizumab	PD1 blocking mAb with Bruton’s TKI	with ACP-196	Phase II	NCT02454179	Advanced HNSCC
Pembrolizumab	Head to head comparison	*vs.* standard treatment (docetaxel/methotrexate/cetixumab)	Phase III	NCT02252042	Recurrent or metastatic HNSCC
Pembrolizumab	Head to head comparison	Pembro+standard treament *vs.* cetiximab+standard treatment	Phase III	NCT02358031	Recurrent or metastatic HNSCC
Pembrolizumab	PD1 blocking mAb	single agent	Phase II	NCT02255097	Recurrent or metastatic HNSCC after CDDP/cetixumab failure
Pembrolizumab	PD1 blocking mAb with IDO1 inhibitor	with INCB024360	Phase I/II	NCT02178722	Advanced or recurrent solid tumors
Pembrolizumab	PD1 blocking mAb with CSF1R blocking mAb	with PLX3397	Phase I/II	NCT02452424	Advanced solid tumors
Pembrolizumab	PD1 blocking mAb	single agent, window-of-opportunity trial before surgery	Phase II	NCT02296684	Advanced but resectable HNSCC
Pembrolizumab	PD1 blocking mAb with B7-H3 blocking mAb	with MGA271	Phase I	NCT02475213	B7-H3^+^ advanced HNSCC
PF-05082566	41BB agonist mAb with PD1 blocking mAb	with Pembrolizumab	Phase I	NCT02179918	Advanced solid tumors
Urelumab	41BB agonist mAb with EGFR targeting mAb	with cetuximab	Phase I	NCT02110082	Advanced/metastatic HNSCC
MEDI4736	PD-L1 blocking mAb with CTLA4 blocking mAb	with or without tremelimumab	Phase III	NCT02369874	Recurrent or metastatic HNSCC
MEDI4736	PD-L1 blocking mAb with STAT3 inhibitor or CXCR2 blocking mAb	with AZD9150 or AZD5069	Phase I/II	NCT02499328	Metastatic HNSCC
MEDI4736	PD-L1 blocking mAb	single agent	Phase II	NCT02207530	Recurrent or metastatic HNSCC
MEDI4736	PD-L1 blocking mAb with HPV E7 expressing Listeria vector	with ADXS 11-001	Phase I/II	NCT02291055	Recurrent or metastatic HPV-associated HNSCC
MEDI4736	PD-L1 blocking mAb with CCR4 blocking mAb	with mogamulizumab	Phase I	NCT02301130	Advanced solid tumors
Tremelimumab	CTLA4 blocking mAb with CCR4 blocking mAb	with mogamulizumab	Phase I	NCT02301130	Advanced solid tumors
Ipilimumab	CTLA4 blocking mAb	with cetiximab and XRT	Phase I	NCT01935921	Advanced HNSCC
Ipilimumab	CTLA4 blocking mAb with B7-H3 blocking mAb	with MGA271	Phase I	NCT02381314	B7-H3^+^ advanced HNSCC
PF04518600	OX40 agonist mAb	single agent	Phase I	NCT02315066	Recurrent or metastatic HNSCC
MDSC targeting trials					
Tadalafil	PDE5 inhibitor	single agent	Phase II	NCT01697800	All stage HNSCC
Therapeutic Vaccines					
VGX-3100 and INO-9012	HPV DNA vaccine	single agent, delivered via IM electroporation, both surgery and CRT arms	Phase I/II	NCT02163057	HPV-associated HNSCC
ADXS 11-001	HPV E7 expressing Listeria vector	with or without αPD-L1 mAb (MEDI4736)	Phase I/II	NCT02291055	HPV-associated HNSCC
ADXS 11-001	HPV E7 expressing Listeria vector	single agent, window-of-opportunity trial before surgery	Phase II	NCT02002182	Stage II-IV resectable HPV-associated OPSCC
CDDP plus VICORYX-2	p16 peptide antigen	with or without Montanide® ISA-51 VG (adjuvant)	Phase I	NCT02526316	HPV-associated HNSCC (p16+)
AlloVax	Whole tumor cell lysate vaccine	with AlloStim adjuvant	Phase I/II	NCT01998542	Metastatic or recurrent HNSCC
Adoptive T Cell Transfer					
Adoptive cell transfer	Ex-vivo TIL expansion with adoptive transfer	combined with lymphodepletion	Phase II	NCT01585428	Metastatic HPV-associated OPSCC
Adoptive cell transfer	Ex-vivo TIL expansion after genetic modification with adoptive transfer	combined with lymphodepletion, viral insertion of a HPV-specific TCR	Phase I/II	NCT02280811	All HPV-associated cancer
Cetuximab-based trials					
Cetixumab	EGFR targeting mAb with standard treatments	with XRT and paclitaxel-poliglumex	Phase I/II	NCT00660218	HPV-negative advanced HNSCC
Cetixumab	EGFR targeting mAb with XRT	XRT	Phase II	NCT00904345	Advanced HNSCC
Cetixumab	EGFR targeting mAb with αCTLA4 mAb with XRT	Ipilumimab and XRT	Phase I	NCT01935921	Advanced HNSCC
Cetixumab	Head to head comparison	cetixumab plus XRT *vs.* CDDP plus XRT	Phase III	NCT01855451	HPV-associated HNSCC
Cetixumab	EGFR targeting mAb with cyclin D inhibitor	with PD0332991	Phase I/II	NCT02101034	Incurable HNSCC
Cetixumab	Head to head comparison	cetuximab *vs.* MEHD7945A	Phase II	NCT01577173	Metastatic or recurrent HNSCC
Cetixumab	EGFR targeting mAb with TLR8 agonist	with VTX-2337, window-of-opportunity trial before surgery	Phase I	NCT02124850	resectable HNSCC
Cetixumab	EGFR targeting mAb with TLR8 agonist and chemotherapy	with VTX-2337 and CDDP+5-FU	Phase II	NCT01836029	Recurrent or metastatic HNSCC

PD1, programmed cell death 1; HNSCC, head and neck squamous cell carcinoma; mAb, monoclonal antibody; IDO1, indoleamine 2,3-dioxygenase 1; CSF1R, colony stimulating factor receptor 1; TKI, tyrosine kinase inhibitor; EGFR, epidermal grwoth factor receptor; CTLA4, cytotoxic T-lymphocyte-associated protein 4; STAT3, signal transducer and activator of transcription 3; PDE5, phosphodiesterase 5; HPV, human papillomavirus; TIL, tumor infiltrating lymphocyte; TCR, T-cell receptor; CDDP, cisplatin; OPSCC, oropharyngeal squamous cell carcinoma; XRT, radiation therapy; TLR8. toll-like receptor 8; 5-FU, 5-flourouracil.

Given recent evidence supporting the role of macrophages in anti-tumor immunity, much pre-clinical work has been done on the ability to functionally repolarize macrophages from a pro-tumor M2 to an anti-tumor M1 phenotype [92,93,94]. As evidence accumulates validating this approach, clinical investigation of agents designed to reprogram macrophages to both limit tumor progression and support the efficacy of standard anti-cancer therapies are warranted.

### 5.2. Targeting Immunosuppressive Cells—T_reg_s

One therapeutic approach that has shown promise in depleting T_reg_s from the tumor microenvironment of mouse models of carcinoma is the use of depleting antibodies targeting CD25 [95]. However, recent clinical reports have demonstrated that depletion of activated, CD25^+^ effector T-lymphocytes occurs along with depletion of CD25^+^ T_reg_s in patients treated with anti-CD25 mAb along with a DC-based tumor vaccine [96]. Another approach involves the use of the PDE5 inhibitor tadalafil that, along with reducing MDSC number and function, appears to reduce the number of circulating and tumor-infiltrating T_reg_s in patients with HNSCC [65,86]. As mentioned above, a phase II trial evaluating the role of tadalafil in HNSCC is underway.

### 5.3. Therapeutic Tumor Vaccines

The goal of a therapeutic vaccine is to introduce whole protein or peptide into a tumor-bearing host and elicit an anti-tumor immune response against TAA or TSA. There are a broad array of platforms that have been utilized to attempt to treat established tumors with therapeutic vaccines, which are summarized in a recent review by Schlom [97]. Common approaches include creating constructs of peptide, which serve as the surrogate TAA or TSA, linked with either adjuvant to initiate an innate immune response and or localizing sequences designed to permit entry inside cells. One major barrier to using specific peptide based vaccines is the requirement of *a priori* identification of TAA or TSA, which is why great excitement exists over the possibility of using therapeutic vaccines to treat HPV-associated oropharyngeal squamous cell carcinoma (OPSCC) with its well-defined viral antigens. This can be overcome when TAA/TSAs are unknown by using whole tumor lysates to *ex vivo* pulse DCs, which include all possible TAA or TSAs. An alternative to using peptide linked to adjuvant involves using bacterial or viral delivery vectors that both deliver peptide to antigen presenting cells and serve as an adjuvant. DNA vaccines are another common approach, but less is understood about how these mechanistically induce anti-tumor adaptive immunity. Several different therapeutic vaccine approaches have shown promise in pilot clinical studies in patients with HNSCC with evidence of induction of anti-tumor immunity in immune correlative studies. These include a melanoma and HPV peptide-based vaccine [98,99], a p53 peptide loaded DC vaccine [100], and most recently a simple multi-peptide and immune adjuvant mixture injected subcutaneously [101]. Based on these results yielding acceptable safety profiles and evidence of induced anti-tumor immunity, many therapeutic vaccine trials are underway.

### 5.4. Ex Vivo Immune Cell Priming with Adoptive Transfer

Another immunotherapy approach involves the *ex vivo* manipulation and activation of a patient’s own immune cells with subsequent adoptive transfer back into the same patient to induce an anti-tumor immune response. This approach was pioneered at the National Institutes of Health. Briefly, a cancer patient’s own T-lymphocytes are extracted and expanded *in vitro* using cytokines and allo-reactive feeder cells. Conversely, the extracted T-lymphocytes can be genetically modified via viral transduction with endogenous or transgenetic T-cell receptors to recognize specific MHC:antigen complexes, or with chimeric antigen receptors which utilize antibody mediated, MHC-independent binding of TAAs or TSAs. After a lymphodepletion approach to rid the patient’s body of competing lymphocytes and immunosuppressive hematopoietic cells, adoptive transfer back into the patient can lead to objective antitumor responses in up to 70% of patients with complete, durable responses in a small subset (reviewed in [102,103]). Small pilot adoptive immunotherapy clinical trials have been reported, mainly on patients with advanced nasopharyngeal SCC [104]. Currently, the Surgery Branch of the National Cancer institute is enrolling patients with advanced HPV-associated OPSCC for adoptive immunotherapy.

### 5.5. Targeting Cancer Cells with Monoclonal Antibodies

Cetuximab is a mAb antibody that targets the extracellular portion of EGFR on the surface of HNSCC cancer cells [105]. FDA-approved for HNSCC in 2006 for concurrent treatment with XRT for advanced HNSCC or as a single agents for recurrent/metastatic HNSCC, and in 2011 for concurrent treatment with CRT for recurrent/metastatic HNSCC, cetuximab serves as an immunotherapy by activating NK cells, which in turn drive antigen-presenting cell maturation and development of adaptive immune responses [106,107]. While many clinical trials investigating the combination of cetuximab with other agents have been designed on the premise that this agent exerts it’s anti-tumor effect via inhibition of signaling downstream of EGFR, many are also investigating the immune mediated effects that may occur.

### 5.6. Therapeutic Antibody Checkpoint Inhibition and Co-Stimulatory Agonists

Given the favorable safety profiles and evidence of durable immune-mediated anti-tumor responses observed in the initial clinical trial to be reported in 2012 [86,87] and reports of dramatic objective responses observed with combination checkpoint inhibition [108], new clinical trials involving immunotherapy have been dominated by those utilizing one or more checkpoint inhibitors. Compared to other forms of immunotherapy that are cumbersome, available at few institutions, and/or often require *a priori* knowledge of a targetable TAA or TSA, checkpoint inhibitors are easy to administer, have few barriers to wide distribution, and non-specifically activate T-lymphocytes. However, evidence to date indicates that checkpoint inhibition activates an existing anti-tumor immune response that is being suppressed by checkpoints expressed within the tumor microenvironment. Evidence that checkpoint inhibition can induce a de novo anti-tumor immune response in a tumor with low baseline immunogenicity is lacking [32,82,109]. Initial reports of single agent PD1 mAb checkpoint inhibition from the Keynote-012 trial have been very promising, with a significant percentage of patients with recurrent and metastatic HNSCC demonstrating PD-L1 positivity (indicative of high baseline tumor immunogenicity) and with >50% of patients demonstrating objective responses to treatment [110]. Trials currently enrolling HNSCC patients combine checkpoint inhibitors with a number of agents designed to enhance the local anti-tumor immune microenvironment such as T-lymphocyte co-stimulatory agonists (CD27 agonist), chemokine receptor blockade (CXCR2, CSF1R and CCR4 blockade) and tumor targeting agents (EGFR targeting mAb and STAT3 blockade).

## 6. Conclusions and Future Directions

While significant strides have been made in our understanding of the role of the immune response in controlling both the development and progression of HNSCC, we are still faced with significant challenges. On one hand, the majority of patients with HPV-associated and a significant portion of patient with carcinogen-associated HNSCC appear to have immunogenic tumors capable of responding to immune-activating therapies such as checkpoint inhibition. But as we gain better insight into the durability of responses observed in these patients, our challenges lie in determining how to enhance the number of patients that respond to such therapies, if possible. Fundamentally, we also need to better understand if poorly immunogenic tumors, with low mutation burden and neoantigens density, can be therapeutically altered to allow the development of an antigen-specific immune response. Clinical trials combining immune therapies together with other types of treatment, such as tumor targeting therapies, will be invaluable to both guide what will be the next generate of “standard of care” HNSCC treatment and to inform the direction of future research.
